# Application of AI Communication Training Tools in Medical Undergraduate Education: Mixed Methods Feasibility Study Within a Primary Care Context

**DOI:** 10.2196/70766

**Published:** 2025-10-24

**Authors:** Chris Jacobs, Hans Johnson, Nina Tan, Kirsty Brownlie, Richard Joiner, Trevor Thompson

**Affiliations:** 1 Department of Psychology, University of Bath, Claverton Down, Bath, BA2 7AY, United Kingdom, 44 01225384176; 2 Bristol Medical School, University of Bristol, Bristol, United Kingdom

**Keywords:** artificial intelligence, technology-enhanced learning, virtual patient, communication skills, simulation

## Abstract

**Background:**

Effective communication is fundamental to high-quality health care delivery, influencing patient satisfaction, adherence to treatment plans, and clinical outcomes. However, communication skills training for medical undergraduates often faces challenges in scalability, resource allocation, and personalization. Traditional methods, such as role-playing with standardized patients, are resource intensive and may not provide consistent feedback tailored to individual learners’ needs. Artificial intelligence (AI) offers realistic patient interactions for education.

**Objective:**

This study aims to investigate the application of AI communication training tools in medical undergraduate education within a primary care context. The study evaluates the effectiveness, usability, and impact of AI virtual patients (VPs) on medical students’ experience in communication skills practice.

**Methods:**

The study used a mixed methods sequential explanatory design, comprising a quantitative survey followed by qualitative focus group discussions. Eighteen participants, including 15 medical students and 3 practicing doctors, engaged with an AI VP simulating a primary care consultation for prostate cancer risk assessment. The AI VP was designed using a large language model and natural voice synthesis to create realistic patient interactions. The survey assessed 5 domains: fidelity, immersion, intrinsic motivation, debriefing, and system usability. Focus groups were used to explore participants’ experiences, challenges, and perceived educational value of the AI tool.

**Results:**

Significant positive responses emerged against a neutral baseline, with the following median scores: intrinsic motivation 16.5 of 20.0 (IQR 15.0‐18.0; *d*=2.09, *P*<.001), system usability 12.0 of 15.0 (IQR 11.5‐12.5; *d*=2.18, *P*<.001), and psychological safety 5.0 of 5.0 (IQR 5.0‐5.0; *d*=4.78, *P*<.001). Fidelity (median score 6.0/10.0, IQR 5.2‐7.0; *d*=–0.08, *P*=.02) and immersion (median score 8.5/15.0, IQR 7.0‐9.8; *d*=0.25 *P*=.08) were moderately rated. The overall Immersive Technology Evaluation Measure scores showed a high positive learning experience: median 47.5 of 65.0 (IQR 43.0‐51.2; *d*=2.00, *P*<.001). Qualitative analysis identified 3 major themes across 11 subthemes, with participants highlighting both technical limitations and educational value. Participants valued the safe practice environment and the ability to receive immediate feedback.

**Conclusions:**

AI VP technology shows promising potential for communication skills training despite the current realism limitations. While it does not yet match human standardized patient authenticity, the technology has achieved sufficient fidelity to support meaningful educational interactions, and this study identified clear areas for improvement. The integration of AI into medical curricula represents a promising avenue for innovation in medical education, with the potential to improve the quality and effectiveness of training programs.

## Introduction

Effective communication is fundamental to high-quality health care delivery, influencing patient satisfaction, adherence to treatment plans, and clinical outcomes [1]. Despite its significance, communication skills training for medical undergraduates often faces challenges in scalability, resource allocation, and personalization. Although traditional methods such as role-playing with standardized patients or observational feedback sessions are effective, they are resource intensive and may not provide consistent feedback tailored to individual learners’ needs in all circumstances [2,3].

Artificial intelligence (AI) offers an innovative solution to address these challenges by simulating realistic patient-provider interactions in controlled, scalable environments. Given their ability to show proficiency in medical knowledge, large language models (LLMs) have been used by medical students in the preclinical phase, as the models aid them in differential diagnosis and provide interactive practice cases to support learning [4]. Recent applications of LLMs in medical education demonstrate expanding capabilities across diverse educational contexts. Advanced LLMs (ChatGPT, Copilot, PaLM, Bard, and Gemini) show comparable performance to students in gross anatomy assessments, indicating potential for educational support in foundational medical sciences [5]. Furthermore, LLMs have shown competitive performance against medical students in specialized topic areas, suggesting potential for supplementing traditional learning approaches [6].

A narrative review of AI in health care communication indicated that these systems have the potential to replicate the complexities of clinical encounters, including interpreting verbal and nonverbal cues, ensuring empathy in responses, and managing dynamic conversational scenarios [7]. Furthermore, communication, as an emotional exchange of information, can be considered a complex system characterized by diversity, nonlinearity of response, interconnectedness of individuals (akin to a neural network), dynamism, feedback orientation, and constant information flow. AI encompasses these facets and can help predict emergent behaviors with machine learning.

AI tools, such as conversational agents and LLMs, can provide health care learners with opportunities to practice communication skills repeatedly and independently while receiving immediate and detailed feedback on performance [8,9]. Simulated virtual patients (VPs) have shown promise in prenatal counseling education, where ChatGPT-generated dialogues increased the realism and variety of patient interactions while maintaining clinical accuracy [10]. In emergency medical services, generative pretrained transformer–based VPs integrated with mixed reality simulation improved the effectiveness of communication training for medical first responders, although technical delays remained a limiting factor [9]. Large-scale implementations have achieved substantial reach, with platforms such as the Geeky Medics Virtual Patient Simulator conducting over 45,000 AI-powered clinical consultations using GPT-3.5 and GPT-4 technologies [11]. However, training needs are substantial yet largely unmet: a previous study reported that 74.8% of medical students wanted structured AI training in medical curricula, but only 26.8% felt competent to inform patients about AI applications [12].

Primary care settings are ideal for communication skills training [13], given the frequent need for effective patient-provider communication to address complex, multidisciplinary health issues. Training future medical professionals in this context requires tools that mirror the intricacies of real-world interactions, including handling diverse patient populations, managing sensitive topics, and navigating the nuances of shared decision-making. AI platforms offer an opportunity to simulate such scenarios with high fidelity while ensuring accessibility and scalability, thus addressing key gaps in current educational approaches [14-16]. Previous studies have used AI in primary care education [17,18]; however, publications using advanced language models to simulate VP encounters remain limited, as the vast majority are discussion papers and viewpoints [19].

To systematically evaluate the educational effectiveness of AI-powered learning tools in health care education, robust measurement frameworks are essential. The rapid proliferation of immersive technologies in medical training, from virtual reality simulations to AI VPs, has created a need for validated assessment instruments that can capture the multidimensional nature of technology-enhanced learning experiences. Recent frameworks, such as the Immersive Technology Evaluation Measure (ITEM) developed by Jacobs et al [20], provide a structured approach to evaluating the educational efficacy of technology-enhanced learning tools and have demonstrated use in conversational AI in medical student evaluation [21]. The ITEM assesses the following domains: engagement, intrinsic motivation, cognitive load, system usability, and postexperience debriefing. These domains are particularly relevant in simulation training with technology, where the fidelity of the simulated interaction as well as the learners’ motivation to engage and ability to reflect on performance are pivotal to achieving meaningful educational outcomes [22]. Applying such frameworks, this study aims to investigate the use of AI technologies in communication training within the primary care context for medical undergraduates.

This study aims to evaluate AI VP technology as a tool to support communication-based learning and to compare this technology with standardized patients used in primary care educational sessions, using quantitative and qualitative analysis. Through quantitative inquiry, the study seeks to evaluate how medical undergraduates perceive the effectiveness and usability of conversational AI, as well as its impact on their confidence and self-reported competence in communication skills. Complementing this, a qualitative analysis explores the experiences of learners using these AI-powered tools, focusing on the challenges and benefits they encounter while practicing communication skills in simulated primary care scenarios. Together, the purpose of these analyses is to provide a comprehensive understanding of the tool’s utility and effectiveness and areas for potential improvement in enhancing communication training for future health care professionals.

## Methods

### Ethical Considerations

Ethical approval was obtained from a regional Independent Research Board, Swindon, United Kingdom (CJ062023). Furthermore, the senior academic staff at the University of Bristol approved the study. Informed consent was obtained from all participants, who were free to opt out at any time. All data were anonymized prior to analysis to ensure privacy and confidentiality. No financial or other compensation was provided for participation.

### Study Design

The study used a mixed methods sequential explanatory design comprising 2 distinct phases: a quantitative phase followed by a qualitative phase. The mixed methods design strengthens the research findings and provides a holistic approach to answer the research aims [23]. The qualitative focus group phase enables a more detailed understanding of the quantitative phase.

**Figure 1.**
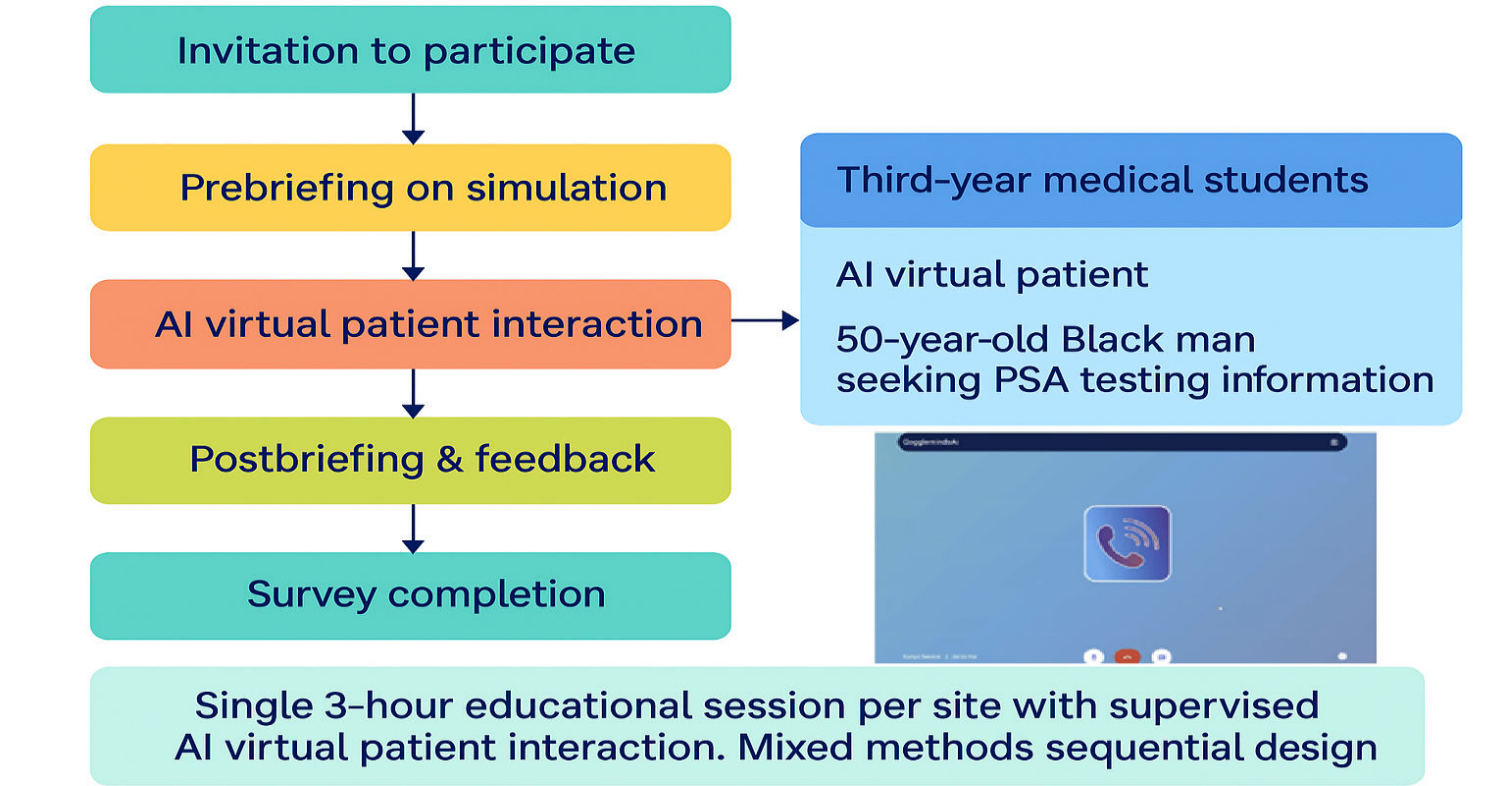
Participant recruitment and study protocol. AI: artificial intelligence; PSA: prostate-specific antigen.

The study sought to provide a nuanced understanding of how AI can enhance communication skills training for medical undergraduates. Primary care training sites were invited to participate via email. Three sites were randomly selected from email responses, with the number limited to 3 for technology oversight purposes. Third-year medical students completing primary care clerkships and general practitioner (GP) facilitators were recruited from 3 selected sites, with 5 students and 1 GP facilitator per site. All approached participants consented to participate (18/18, 100% response rate). The participating students (n=15) represented 6% of the total third-year medical student population (N=240), with GP facilitators totaling 3 participants. Students in their third year of study were selected, as the third-year curriculum focuses on primary care communication skills. Practicing doctors were included because they represent the educator demographic who would implement AI VP technology in clinical teaching. Their participation provided stakeholder perspectives on feasibility, usability, and educational value from the instructor viewpoint. Educational sessions at the primary care sites lasted 3 hours and were conducted as single-exposure experiences at each site. The theme of the day was evaluating urological cancer, and students interacted with the AI VP (20 min per consultation) only during these supervised sessions, with no access provided outside the research period. Standardized materials were provided to the facilitators to conduct a prebriefing on simulation and important aspects of communication. Learning objectives included developing shared decision-making communication skills for prostate cancer screening discussions; explaining the benefits and limitations of prostate-specific antigen (PSA) testing, including the concepts of specificity and sensitivity; exploring patient concerns and health anxieties; applying evidence-based counseling techniques for screening decisions; and addressing patient questions about ethnicity-related cancer risks. Furthermore, the facilitator at each site conducted a postbriefing on student performance on consultations. The students and facilitators were invited to attend 3 focus group discussions following their participation in AI simulation and completion of the initial survey. Figure 1 outlines the procedure and shows a screenshot of the display seen by participants. Survey methods are reported using the Consensus-Based Checklist for Reporting of Survey Studies (CROSS; Checklist 1).

### Technical Implementation

The VP was constructed using OpenAI’s GPT-3.5-turbo LLM accessed via RESTful application programming interface (API) end points. The system was hosted on the Firebase cloud infrastructure (Google LLC), using Firebase Authentication for secure user access, Firestore database for session management, and Firebase Hosting for web application deployment. All communications with the OpenAI API were encrypted using HTTPS protocols. The language model was configured with specific parameters to optimize conversational flow and educational authenticity. API requests used a temperature setting of 0.7 to balance response creativity with consistency.

The AI VP was programmed with a comprehensive system prompt defining the patient persona as a 50-year-old Black man seeking information about PSA testing. The prompt included specific instructions to maintain character consistency, use an appropriate level of medical knowledge for a lay person, express realistic health concerns and anxieties, and avoid breaking the illusion of being a real patient (see Multimedia Appendix 1 for more information on the prompt). Conversational context was maintained through session-based memory management. Students accessed the AI VP through a secure web-based interface featuring a conversational chat window with dual input modalities. The interface supported both typed text responses and voice input captured via Web Speech API (Google LLC) for speech-to-text transcription. User authentication was implemented through Firebase Authentication to ensure secure access and session isolation between participants. Student speech was captured using browser-based microphone interfaces and converted to text using the Web Speech API configured for British English language recognition. AI responses were converted to natural speech using the ElevenLabs text-to-speech synthesis service, configured with a professional male voice model emulating a London accent. Prior to deployment, the research team extensively tested the AI VP by interacting with the VP as a doctor to ensure consistent character portrayal, appropriate medical knowledge representation, and reliable technical performance. Content validity was achieved by testing, and ecological validity was sought by real-world deployment in primary care settings. A video example of this system is available in Multimedia Appendix 2.

### Survey Instrument and Focus Group Development

The survey used an abridged version of the ITEM, which assesses 5 domains of experience in a health care education context using technology-enhanced learning in simulation [24,25]. The engagement subdomain included 2 questions related to the realism (fidelity) of the experience. The measure was developed on the basis of the Model for Immersive Technology in Healthcare Education (MITHE), which proposes that the learners' perception of the usability of technology is integral to experiential learning [20]. System usability was measured as learners’ perceptions of technology ease of use (using adapted System Usability Scale items), recognizing that these perceptions are influenced by individual learners’ characteristics such as prior technology experience and adaptability to novel interfaces. The study team reviewed the full ITEM questionnaire (40 questions) and reduced the number of questions to 12, which is supported by Jacobs and Rigby’s [24] work on measure development, minimizing the effect on internal consistency and maintaining construct validity. Domain scores were calculated by summing the individual item responses within each ITEM domain: fidelity (2 items, range 2‐10), immersion (3 items, range 3‐15), intrinsic motivation (4 items, range 4‐20), debriefing psychological safety (1 item, range 1‐5), and system usability (3 items, range 3‐15). All items were rated on a 5-point Likert scale with a consistent scoring direction. The Intrinsic Motivation Inventory was used to assess the perceived interest, learning value, and user competence, as proposed by the self-determination theory [26]. The survey was designed by medical tutors, and the construct was derived from the work of a consortium of simulation specialists on using simulation educational technology using the Delphi methodology [25].

In the subsequent focus group sessions (n=3), the interview data were enhanced by 4 open-ended questions in the initial survey to explore the authenticity, realism, and potential learning value of the interaction and to compare AI to human patients. Each focus group was facilitated and led by a medical tutor (KB), and 5 areas of questioning were developed by the study team. An interview guide with topics on educational value, realism, technical aspects, further development, and future applications was created, allowing for deeper probing through conversation (see Multimedia Appendix 3 for topic guide). Focus group interviews were conducted after the AI interaction, and the end data produced were obtained from participants from AI VP groups. Additionally, each focus group included a medical tutor to enrich the perspectives, with a facilitator encouraging student input to minimize the power differential this created.

### Data Analysis

#### Quantitative Component

Quantitative data obtained through the survey instrument ITEM were analyzed using both descriptive and inferential statistical methodologies, using nonparametric methods suitable for Likert scale data. Statistical analysis was performed using R software (version 4.3.2; R Foundation for Statistical Computing) [27]. The primary metrics used to summarize the responses for each domain (fidelity, immersion, intrinsic motivation, debriefing, and usability scores) were the median and IQR, as these are the most appropriate measures of central tendency and variability for ordinal data. This inference provides an accurate representation of participants’ response without assuming a normal distribution. The median rating for each domain was standardized as a percentage of the maximum possible score to allow for fair comparisons of ratings. Given the small sample size, inferential statistical analysis was limited to exploratory purposes. Single group nonparametric statistical analysis was conducted using 1-sample Wilcoxon signed rank tests comparing participant domain scores against neutral response values (domain midpoints) [28]. Neutral values represent a theoretical reference point where there is a lack of prior experience and represent a conceptual no-effect condition [29]. This approach was selected due to the ordinal nature of Likert scale data and small sample size precluding parametric assumptions. Effect sizes were calculated using Cohen *d* conventions (0.2=small, 0.5=medium, 0.8=large effect) to assess practical significance alongside statistical significance. Statistical significance was set at *P*<.05 for this exploratory analysis, with no corrections applied for multiple comparisons given the pilot study design.

#### Qualitative Component

Open-ended survey responses were uploaded to NVivo software (version 12; QSR International Pty Ltd) for an initial content analysis. A thematic content analysis was performed to investigate the participants’ experience of using an AI VP to simulate a primary care consultation. The thematic analysis was performed using a hybrid inductive-deductive approach guided by the MITHE framework and ITEM domains while remaining open to emergent themes. The focus group data were analyzed by 1 author (KB), and the open-ended response codes and themes were explored by 2 other authors (CJ and HJ). The thematic analysis was conducted following the 6-step framework outlined by Braun and Clarke [30], encompassing getting familiarized with the data, generating initial codes, searching for themes, reviewing themes, defining and naming themes, and producing the final report. A provisional codebook was developed from theoretical constructs and then iteratively refined through independent dual coding by 2 authors (CJ and HJ). Codebook development used a collaborative shared document approach with interrater agreement, and discrepancies were resolved through a subsequent consensus discussion. Finally, 2 authors (CJ and HJ) refined and revised the datasets to capture the overall narrative and major themes. Furthermore, the open-ended question dataset was analyzed on the basis of a study by Braun et al [31], who suggest that a reduced transcript length can be both concise and informative, retaining the unguarded perspectives of participants.

## Results

### Demographics

The survey respondents included third-year clinical medicine undergraduate students from a UK medical school. Of the 18 participants, 15 were medical students (83%), and 3 (17%) were practicing doctors in primary care and group tutors. No participants had prior experience interacting with an AI-simulated patient.

### Quantitative Results

There were no missing data from the 18 participants.

**Figure 2.**
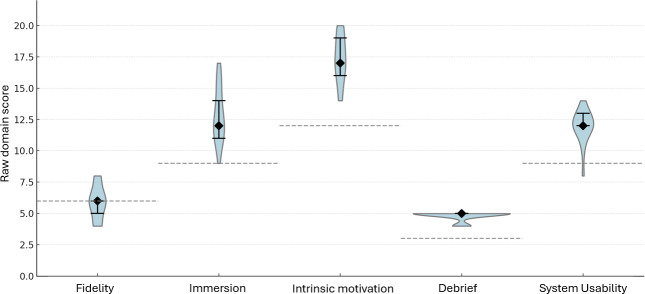
Violin plot of raw domain scores versus a neutral benchmark.

A quantitative survey assessed the perceived effectiveness and usability of the AI tool and the medical undergraduates’ engagement with the tool. Results are summarized in Table 1, and the results of the 5 domains are standardized in Figure 2.

**Table 1.** Summary of the AI^a^ educational domain ratings.

Domain	Raw median score/maximum possible score (IQR)	Difference between the median and neutral scores	Standardized median score/5.0 (IQR)	Median score as percentage of maximum possible score (%)	Effect size (*d*)	*P* value^b^	Context^c^
Fidelity	6.0/10.0 (5.2-7.0)	0.0	3.0 (2.6-3.5)	60.0	–0.08	.02	Realism of AI virtual patient communication
Immersion	8.5/15.0 (7.0-9.8)	0.5	2.8 (2.3-3.3)	56.7	0.25	.08	Participant engagement and immersion
Intrinsic motivation	15.5/20.0 (15.0-18.0)	4.5	4.1 (3.8-4.5)	82.5	2.09	<.001	Internal motivation and learning potential
Debriefing	5.0/5.0 (5.0-5.0)	2.0	5.0 (5.0-5.0)	100.0	4.78	<.001	Quality and safety of reflective discussion
System usability	12.0/15.0 (11.5-12.5)	3.0	4.0 (3.8-4.2)	80.0	2.18	<.001	Ease of use and accessibility of the platform
Total	47.5/65.0 (43.0-51.2)	9.5	3.6 (3.3-3.9)	73.1	2.00	<.001	Combined score across all domains

a AI: artificial intelligence.

b *P* values derived from the Wilcoxon signed rank test comparing each domain’s score to a neutral reference point.

c Context summarizes what each domain aims to measure in relation to the AI-enhanced learning activity.

The fidelity or realism of the AI communication had a moderate median score of 6.0 (IQR 5.2‐7.0), of a maximum possible score of 10.0. Furthermore, the median score for immersion was 8.5 (IQR 7.0‐9.8), with a maximum possible score of 15.0. The difference between the median score and a neutral response comparator (ie, median difference) was equivalent in both domains (fidelity: median difference=0.0; *P*=.02; immersion: median difference=0.5; *P*=.08).

Intrinsic motivation had a high median score of 15.5 (IQR 15.0‐18.0), with a maximum possible score of 20.0. There was a significant difference compared to the neutral response, with a large effect size (median difference=4.5; *d*=2.09, *P*<.001).

A universally high score was reported for the psychological safety of interacting with an AI VP during simulation debriefing (median 5.0, IQR 5.0‐5.0). There was a significant difference compared to the neutral response, with a large effect size (median difference=2.0; *d*=4.78, *P*<.001).

System usability was highly rated, with a median score of 12.0 (IQR 11.5‐12.5) on a maximum possible score of 15.0, which showed a significant positive difference from the neutral response, with a large effect size (median difference=3.0; *d*=2.18, *P*<.001).

The total measure score was high, with a median of 47.5 (IQR 43.0‐51.2), and showed a significant positive median difference compared to the neutral response, with a large effect size (*d*=2.00, *P*<.001).

### Qualitative Results

**Figure 3.**
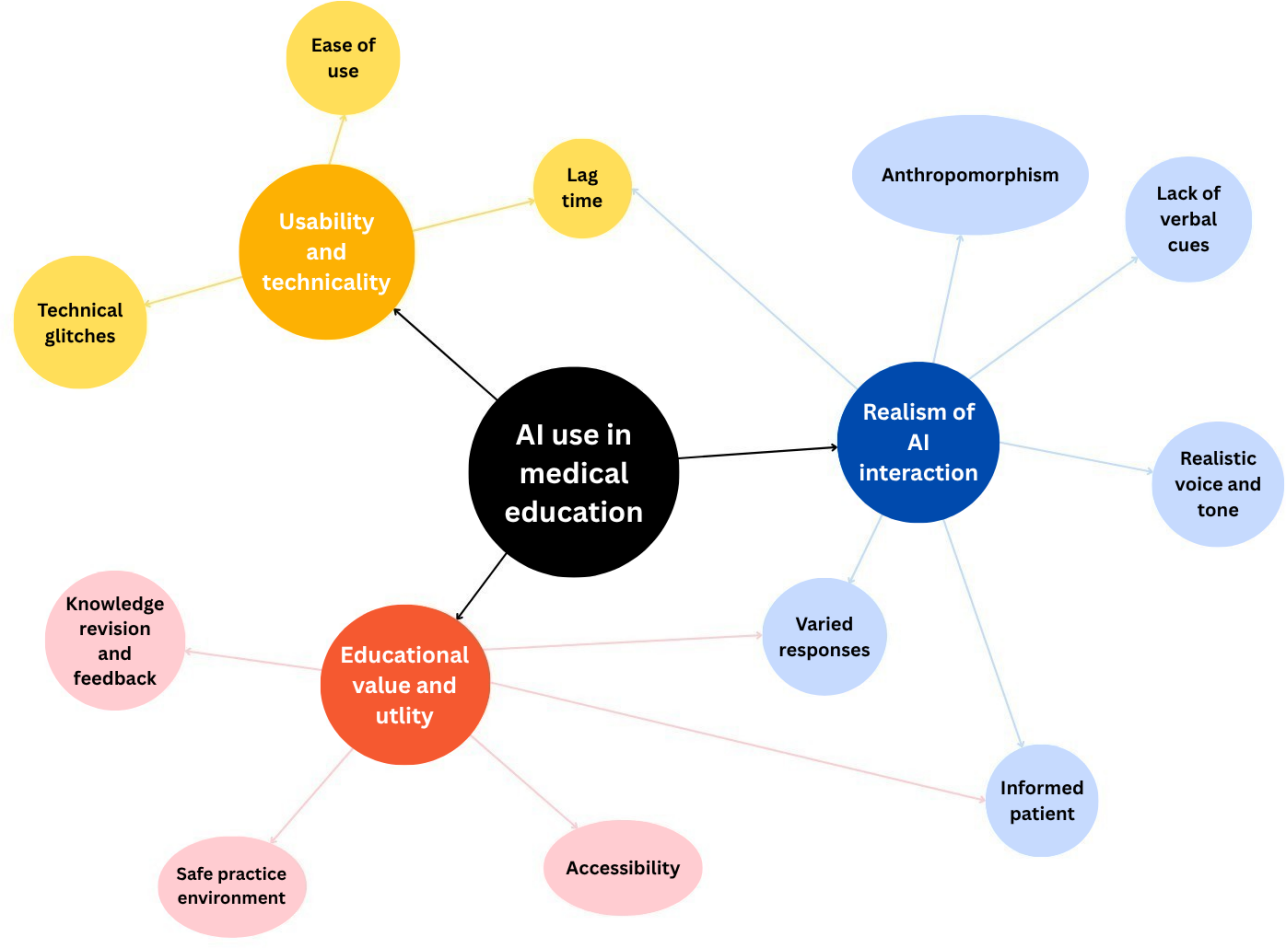
Thematic map displaying the interlinking themes and subthemes. AI: artificial intelligence.

Following a collective review of the responses of 18 participants to the focus group questions and open-ended questions, 3 overarching themes were identified, supported by 11 subthemes (Table 2 and Figure 3). Convergence of these themes and integration with quantitative results are described later under Key Findings and Implications in the Discussion section.

**Table 2.** Qualitative summary of the major themes and subthemes identified from responses to open-ended survey questions and focus group interviews.

Theme and subtheme	Description	Illustrative quote (participant number; quote ID)
Usability and practicality		
Time lag	Significant pauses disrupted the natural flow of conversation and reduced realism.	“Long pauses between questions. Hard [*sic*] felt like emotionless answers.” (P3; Q1) "The pause between question and answering made it difficult for the consultation to flow and didn’t feel it replicated a ‘real’ consultation.” (P6; Q2)
Technical glitches	System crashes or repeated questions caused frustration and disrupted learning.	“There were a lot of faults and hiccups as we did it but wasn’t too bad overall.” (P12; Q3) “There was also some glitches that required questions to be asked multiple times or for the consulting student to begin again.” (P17; Q4)
Ease of use	The technology was generally easy to access and use.	“The technology [is] straightforward to access and use.” (Focus group 1; Q5)
Realism of AI^a^ interaction		
Anthropomorphism	Some interactions felt mechanical, with reduced empathy and natural conversational flow.	“It felt very robotic with a lot of miscommunication.” (P12; Q6) “Robotic answers and there was a delay in responding.” (P16; Q7) “It felt like a realistic conversation.” (Focus group 2; Q8)
Informed patient	AI responses were either too short or overwhelming with unnecessary details.	“The AI gave more information unprovoked/not asked for than a patient normally would.” (P17; Q9) “The patient was very well informed.” (P13; Q10) “Gave very scientific responses.” (P10; Q11)
	AI used technical language inappropriate for a simulated patient role.	“The AI was using medical jargon that you wouldn’t normally expect a patient to use.” (P17; Q12)
Lack of nonverbal cues	The absence of body language and facial expressions limited realism and engagement.	“Also not being able to see the AI’s body language.” (P16; Q13)
Educational value and utility		
Realistic voice and tone	AI’s voice and tone were seen as realistic and contributed to a sense of authenticity.	“AI voice was human-like, but the answers were a bit too succinct.” (P5; Q14) “Realistic voice.” (P13; Q15) “I think the voice was very realistic, the time delay wasn’t too much of an issue.” (P18; Q16)
Varied responses	AI responses were sometimes unexpected, adding a layer of realism.	“The AI patient gives unpredictable answers and asks questions like a patient would.” (P16; Q17) “Was really impressed by how the AI bot could interpret conversational language and respond appropriately.” (P9; Q18)
	AI responses were repetitive, leading to a lack of conversational depth.	“There were limited responses however and we got a sense of déjà vu with some of the responses.” (P9; Q19) “It answered some questions that we didn’t ask.” (P17; Q20)
Knowledge revision and feedback	AI reinforced clinical knowledge effectively and provided a platform for self-assessment.	“Very useful knowledge checking in a clinical setting.” (P1; Q21) “The AI can ask questions which helps with the learning.” (P7; Q22) “Good to learn clinical knowledge.” (P4; Q23)
Safe practice environment	AI provided a low-risk space for students to practice and make mistakes without judgment.	“It would be very useful as a revision tool to check how we as students present knowledge/consult with patients.” (P5; Q24) “This was a good tool for practicing giving patients information in a safe space.” (P9; Q25)
Accessibility	The ability to use AI for practice outside formal clinical settings was highly valued.	“Allows us to practice at home.” (P10; Q26) “It would be really useful to have access to it at home to practice with.” (P17; Q27)

a AI: artificial intelligence.

#### Theme 1: Usability and Practicality

The AI VP interface demonstrated mixed usability outcomes across 3 key domains. Response delays of approximately 2‐3 seconds consistently disrupted the natural flow of conversation and reduced perceived realism (Q1 and Q2), impacting participants’ ability to maintain authentic consultation dynamics. System instability presented additional challenges, with technical faults requiring question repetition or consultation restarts (Q3 and Q4), interfering with the intended focus on communication skills development. Despite these technical limitations, participants found the interface straightforward to access and operate without requiring technical support or extensive instruction (Q5), facilitating smooth adoption across focus groups.

#### Theme 2: Realism of AI Interaction

Realism emerged as the most debated aspect among participants, with varied perceptions across multiple dimensions of the VP experience. Participant perspectives on the AI’s humanlike qualities were mixed: while some participants found the interactions mechanical and emotionless (Q6 and Q7), focus groups generally perceived the overall experience as reasonably authentic (Q8). The AI’s knowledge level created divergent opinions, with some viewing the detailed, scientific responses as unrealistic for typical patients (Q9, Q10, and Q11), particularly when medical jargon was inappropriately used (Q12). The audio-only format limited realism owing to the absence of visual and nonverbal communication cues (Q13), reducing engagement and authenticity compared to face-to-face consultations. However, participants praised the AI’s voice quality and tone as contributing to authenticity (Q14, Q15, and Q16), supporting the simulation’s credibility as a telephone consultation format. The AI’s ability to provide unpredictable responses and interpret conversational language enhanced perceived authenticity (Q17 and Q18), although repetitive responses occasionally created a sense of déjà vu, diminishing the conversational depth (Q19 and Q20).

#### Theme 3: Educational Value and Utility

Participants recognized significant educational potential across 3 key areas that could enhance medical training. The AI effectively reinforced clinical knowledge and provided opportunities for self-assessment (Q21, Q22, and Q23), with participants valuing the AI’s ability to prompt a discussion of clinical concepts they might not have considered. The virtual environment offered a psychologically safe practice space without the risk of patient harm or judgment (Q24 and Q25), enabling students to experiment with consultation approaches and make mistakes without consequences. Participants particularly valued the potential for accessible, asynchronous learning outside formal clinical settings (Q26 and Q27), representing aspirational use for home-based practice and revision, although access was limited to supervised study sessions during the research period.

## Discussion

This mixed methods study explored the application of AI communication training tools in medical undergraduate education within a primary care context. The findings indicate that AI VP encounters can enhance communication skills training by providing a scalable, accessible, and realistic simulation environment.

### Key Findings and Implications

Participants reported moderate levels of perceived realism (fidelity score 6/10), with the voice quality achieving authenticity but technical limitations, such as response delays and overly clinical language, reducing overall fidelity. Despite these limitations, the AI VP demonstrated sufficient realism for educational engagement, as evidenced by the high intrinsic motivation score (16.5/20.0) and positive learning value feedback.

In particular, the results demonstrated high levels of perceived effectiveness, usability, and psychological safety among participants. High median scores for intrinsic motivation and system usability were particularly notable, indicating strong engagement and ease of use. These findings align with previous research suggesting that AI tools can effectively support medical education by providing immediate feedback and opportunities for repeated practice [32,33]. The high intrinsic motivation score suggests that students found the AI tool engaging and beneficial for their learning, which is crucial for the adoption of new educational technologies [34]. Learning motivation can be seen as a mediator between technological acceptance and self-efficacy on the task [35,36]. Importantly, AI technologies create a dynamic and adaptive learning ecosystem that tailors educational experiences to individual preferences, enhancing engagement and effectiveness.

Immersion scores (median 8.5/15) aligned with prior ITEM validation research demonstrating that computer screen–based educational experiences typically achieve moderate immersion levels [37]. These scores may reflect the inherent limitations of a 2D interface rather than deficiencies of AI VPs. Importantly, prior studies have found equivalent learning outcomes between high-immersion virtual reality and 2D screens, suggesting moderate immersion may be optimal because it avoids excessive cognitive load while maintaining educational presence.

Technological acceptance on the basis of reasoned action was first proposed as the technology acceptance model [38], which is used to explain the association between acceptance of a computer system and the behavioral intention thereof. System usability within human-computer interaction is critical in the use of technology [39]. The MITHE framework provided an appropriate theoretical basis for our AI VP evaluation, as conversational AI creates immersive learning experiences through cognitive and social engagement rather than visuospatial immersion. System usability within MITHE encompasses both learner perceptions of technology ease of use and individual learner characteristics that influence technology adoption, both of which were evident in our participants’ varied responses to the AI VP interface. Furthermore, in the context of technological learning, this means that students who find technology-based learning activities inherently interesting are more likely to engage deeply and persistently. Thus, there is a paradigm of interrelating concepts between engagement and usability. The AI tool enabled both to be established and demonstrated an educational value to students.

Realism in simulation refers to the degree to which a simulation accurately represents real-life scenarios. It involves creating an environment that closely mimics actual clinical situations, allowing learners to engage in tasks and decision-making processes as they would in real clinical settings. Realism is necessary for ensuring that the skills and knowledge acquired during simulation training are transferable to real-world practice [14,40]. Fidelity in simulation is the extent to which the simulation replicates the real-world environment and experiences. It encompasses various dimensions, such as physical, conceptual, and psychological dimensions [41]. The degree of fidelity in this pilot study was reported as moderate by participants. Participants highlighted issues such as time lag, technical glitches, and the lack of nonverbal cues, which impacted the perceived fidelity of the AI interactions. The AI VP provided a naturalistic voice, which was the foundation for a humanlike experience. However, the psychological fidelity was impacted by the extent to which learners could emotionally and cognitively engage with the AI as if they were in a real situation. High psychological fidelity scenarios have the potential for students to develop not only their communication skills but also their empathy and bedside manner.

While AI VPs may not have a physical presence, their interactions can be designed to closely replicate real patient encounters. This includes realistic voice modulation, appropriate use of medical terminology, and the ability to simulate complex medical scenarios. In this study, the scenario required the AI VP to simulate a patient asking a doctor questions, leading to a balanced discussion on PSA testing. Participants appreciated the AI’s ability to simulate informed patient interactions, which enhanced their clinical knowledge and decision-making skills. However, some noted that the AI’s responses could sometimes be overly detailed or robotic, detracting from the realism of the simulation. This feedback suggests that future iterations of AI VP should aim to balance informativeness with a natural conversational flow. The ability of the AI to provide detailed responses was seen as both a strength and a limitation, indicating the need for a more nuanced approach to AI response generation.

AI models, especially advanced ones such as LLMs, can understand the context of a prompt. This means they can interpret the nuances of a question or statement and generate responses that are contextually appropriate. For example, if a medical student asks about symptoms of a specific condition, the AI model can provide detailed information relevant to that condition [42]. This is somewhat incompatible with the patient perspective of lacking the understanding of a problem because of which they consult with a physician. A dynamic interaction improves realism, and the varied responses by the AI VP provided users with adaptive and personalized responses. Advanced AI models can simulate emotions and empathy in their responses [7,43]. For example, if a student is practicing delivering bad news to a patient, the AI can respond with appropriate emotional cues, such as expressing concern or asking for clarification, to mimic a real patient’s reaction. A narrative synthesis of learning empathy through simulation suggests that simulation is an appropriate method to teach empathy to preservice health professional students [44].

In a pilot study among medical students, earlier LLM models, for example, GPT-3.5, that generated text-only responses were reported to create a plausible experience for students. The analysis revealed that most answers provided by the LLM were medically plausible and in line with the illness script [45]. Vaughan et al [46] recently reported that LLMs can create realistic simulation examples following text-based review. Furthermore, the accuracy, relevance, and structure of the AI programs benefit review prior to adoption in medical education settings [47].

The thematic analysis also revealed that participants valued the safe practice environment provided by the AI VPs. The ability to practice communication skills without the risk of harm to real patients was seen as a significant advantage, supporting the use of AI in medical training [48]. Additionally, the accessibility of the AI tool, allowing for asynchronous learning, was highlighted as a key benefit, particularly for students needing flexible study options [49]. Cognitive presence and social presence are essential for a comprehensive learning experience. While asynchronous learning environments offer flexibility to accommodate individual schedules, they also present challenges in enhancing cognitive and social presence. Despite an informative and realistic interaction, this aspect of learning may have been impacted, as participants assigned immersion a moderate median score. Social presence is the ability of learners to project themselves socially and emotionally in a learning environment. It involves the sense of being “there” and being able to interact meaningfully with others. The illusion of presence—that is, a student wholly experiencing the interaction as if they are a doctor talking to a real patient—may be broken by a simple time lag in response [50].

### Educational Value and Future Applications

The study’s findings suggest that AI-powered VPs have substantial educational value, particularly in enhancing communication skills in primary care settings. The positive feedback on knowledge reinforcement and the potential for personalized feedback indicates that AI tools can complement traditional training methods, providing a more comprehensive learning experience. The ability to receive immediate, detailed feedback from the AI was particularly valued by participants, highlighting the potential of AI to support continuous improvement in communication skills, contributing to the broader argument of adoption of AI in medical education curricula. Further advances in AI computational complexity with a reduced time lag in response and authenticity of character will further improve learner experience. There are numerous use cases beyond this pilot study, including patient standardization for objective structured clinical examinations, simulated consulting, advanced manikin simulation with realistic patient response, and procedural skills communication feedback. Integrating machine learning into the review of a participant’s behavior and response to AI VP could add a degree of automation to medical and allied medical licensing examinations. Model variations allow for changes in VP characteristics; however, model stability and sensitivity need further investigation. Future development should prioritize technical infrastructure to minimize response delays using API optimization, advanced prompt engineering to achieve more natural conversational patterns, and adaptive information delivery systems that balance patient authenticity with educational value. Conversational analysis would be an important step to evaluate the clinical narrative and naturalness. Eventual integration of visual avatars and enhanced voice prosody could address nonverbal communication limitations while maintaining the accessibility advantages of current screen-based implementation.

### Strengths and Limitations

To our knowledge, this is the first study to use an advanced, realistic voice on a recent LLM model, with the aim to assess simulation realism for students for use in practicing consultation skills. The context of primary care consulting that focused on discussing a case of PSA testing additionally tested the AI in a conversation that helped students develop their cognitive reasoning skills. The use case provides evidence for future work in assessing the accuracy of responses and AI feedback on the consultation via transcript analysis.

Being a pilot study, this study was underpowered, and the results demonstrating statistical significance (*P*<.05) with large effect sizes need to be interpreted with caution. This study was conducted at 3 randomly selected sites with a small sample of students (n=15) representing 6% of the third-year cohort (N=240), which may limit the generalizability of findings to the broader medical student population despite the random site selection. Furthermore, the measures were abridged versions of validated questionnaires. This study did not aim to analyze the content validity; however, construct validity was assumed from prior work in this field. Additionally, this was an observational study, and a comparison of human consultation was not conducted, which is important for future work. The assumption of high intrinsic motivation and other domains requires further comparison to standard learning methods such as using actors.

Learning objectives focused on shared decision-making communication skills for prostate cancer screening discussions. This feasibility study assessed the perceived educational value of an AI VP through ITEM scores and qualitative feedback rather than a formal pre- and postcompetency assessment, representing a limitation. Future research should incorporate validated communication skills assessments to quantify learning outcomes objectively.

Focus groups enable the inclusion of a social element into the development of ideas on a topic; however, the presence of a tutor during the data collection may have influenced the responses by students, even when the facilitator is attuned to this and adapts questions to promote collective contribution.

### Conclusion

The research aimed to explore the practical application of an AI VP tool in preparing future health care professionals for real-world patient interactions. AI VP technology shows promising potential for communication skills training despite the current limitations in realism. While it does not yet match human standardized patient authenticity, the technology achieved sufficient fidelity to support meaningful educational interactions. Furthermore, the study identified clear areas for improvement. The integration of AI into medical curricula represents a promising avenue for innovation in medical education, with the potential to improve the quality and effectiveness of training programs. Future research focusing on comparison with medical actors in assessment and AI-generated participant score will assist in strengthening the argument for AI integration into health care education.

## Supplementary material

10.2196/70766 Multimedia Appendix 1 System prompt for artificial intelligence (AI) virtual patient.

10.2196/70766 Multimedia Appendix 2 Video example of the artificial intelligence (AI) system.

10.2196/70766 Multimedia Appendix 3 Artificial intelligence (AI) virtual patient focus group facilitator topic guide.

10.2196/70766 Checklist 1 Consensus-Based Checklist for Reporting of Survey Studies (CROSS).

## Abbreviations

AI: artificial intelligence
API: application programming interface
CROSS: Consensus-Based Checklist for Reporting of Survey Studies
GP: general practitioner
ITEM: Immersive Technology Evaluation Measure
LLM: large language model
MITHE: Model for Immersive Technology in Healthcare Education
PSA: prostate-specific antigen
VP: virtual patient
