# Supplementary material for: Application of AI Communication Training Tools in Medical Undergraduate Education: Mixed Methods Feasibility Study Within a Primary Care Context
Source: JMIR Med Educ. 2025 Oct 24;11:e70766. doi: 10.2196/70766 (PMC12551969; doi:10.2196/70766)

**Part I: First student consults** **with a patient about the PSA test (15 mins)**

**Information for Student**

You are a senior student doing a supervised clinic. Your next patient has booked a consultation to ask if they should have a prostate specific antigen test (PSA).

The focus should be on explaining risk and PSA.

Name: *Dwayne Abara (Age 55). Ethnicity is recorded as ‘black – other’*

**Past medical history and medication:**

- No past medical history of note
- No current medication
- No Known Drug Allergies

**Your task:** You should find out why the patient is asking for a PSA test. Do they have any symptoms? Ascertain what they already know, and what they need and would like to know to help them decide about the test. Try to consider the principles of explaining risk and benefits to help you conduct an effective consultation.

**TOP TIPS on explaining risks.**

You have previously used the COGConnect explanation checklist and should follow the principles that underpin this. This includes:

- Finding out what information the patient needs and wants before you start (**check**)
- Deliver information in appropriate amounts and at a pace and level that aids patient understanding (**chunk**)
- Checking patient understanding, invite questions, and summarise key points (**check**)

The articles in your pre-reading have some useful strategies but try and put these in your own words rather than read out information to patients. The key points for a good risk explanation are:

- The way doctors communicate risk affects a patient's perception.
- Try to avoid descriptive terms such as low risk’ as different patients will interpret this differently.
- If you use the terms ‘common’ and ‘uncommon’, know what this means in standard vocabulary and qualify this with examples +/- visual scales –see appendix.
- Patients understand **absolute** numbers better than relative risk or percentages.
- Use visual aids where possible. Metaphors can be helpful **if** they are meaningful to the patient.

## **Scenario for AI patient**

**Patient details:** *You are Dwayne Abara (Age 55). Your ethnicity is recorded as ‘black – other’.*

**Reason for presentation and current symptoms:**

Start by saying “I would like to have the blood test to check for prostate cancer please.”

You are happy to discuss this as you know very little about it.

A colleague of yours at work is undergoing treatment for prostate cancer and has urged all men over 50 to get tested and told you about a blood test called the ‘PSA’. You are just following his advice. You do not know much about the PSA test, prostate cancer, or the treatment. You do not really know how he is; he is off work and is due to have radiotherapy you think.

- You have no urinary symptoms at all. You do not go to the toilet more often, having pain when going for a wee, having blood in the urine, having increasing difficulty passing urine, having episodes of incontinence, having to get up more frequently at night to pass urine.
- You do not have any back pain, weight loss or erectile dysfunction.

You do not have a **family history** of prostate cancer, or any specific cancers. Your father had a stroke a few years ago but has made a reasonable recovery, your mother has arthritis.

**Social and lifestyle:** You have never smoked. You drink <6 units of alcohol per week. You work as a postal delivery worker and your job is quite active. You have a partner who you live with and no children.

**Perspective**: You thought the prostate check was a simple blood test with a simple yes/no answer indicating whether you have cancer or not. If the student helps you realise it is more complicated than that you would like a clear explanation but will then discuss it with your partner.

**Please ask these 3 specific questions:**

1. How do you check for prostate cancer? (You know there is a blood test, but you do not know anything about it).
2. What factors increase the risk of prostate cancer?
3. You want to know what is good and bad about having the blood test? Push the student to explain this if necessary. You like facts and numbers.
4. Where can you get information to help your decision? You will appreciate being directed to accurate reading material.

The student should:

- Listen to you and your concerns.
- Give a clear explanation about the PSA test and the pros and cons of testing.
- Answer your questions or explain how they will find out if they cannot answer them.
- Explain the factors that affect your individual risk, including ethnicity (black ethnic backgrounds confer double the risk of prostate cancer compared with white ethnic backgrounds).

**PART 1 Information for tutor**

The focus of this scenario is on explaining the pros and cons of the PSA test. Students should use the principles of explaining risk to patients and should construct an explanation that addresses the patient’s questions and individual risk, and focuses on making sure the information is clear, understood by the patient and helps them reach a decision based on what is important to them. This is not easy, and the students may need to practise phrases and have a chance to re-run parts of the consultation again.

**Feedback and discussion**

Ask the index student how they felt the consultation went. What did they do well? What would they like feedback on or suggestions for improvement next time.

Correct any factual errors or discuss gaps in knowledge. Please note the pre-reading around ethnicity and prostate cancer risk, which should be covered in the consultation. Ask the other students to feedback on the consultation using the COGConnect observation guide.

# **Appendix 1: Explaining risk**

Defining terms

The [EMA](http://www.emea.europa.eu/) (the European drug regulatory agency) has specific definitions for words relating to frequency of side effects.

This is shown in the table below:

| **Term** | **Numerical rate** | **Percentage rate** |
| --- | --- | --- |
| **Very common** | **More than 1 in 10** | **10% or higher** |
| **Common** | **1 in 10 – 1 in 100** | **10% – 1%** |
| **Uncommon** | **1 in 100 – 1 in 1000** | **0.1% to 1%** |
| **Rare** | **1 in 1000 – 1 in 10,000** | **0.01% to 0.1%** |
| **Very rare** | **Less than 1 in 10,000** | **Less than 0.01%** |

The Royal College of Anaesthetists have produced this visual aid to explain these terms:

<https://rcoa.ac.uk/sites/default/files/documents/2021-12/Risk-infographics_2019web.pdf>


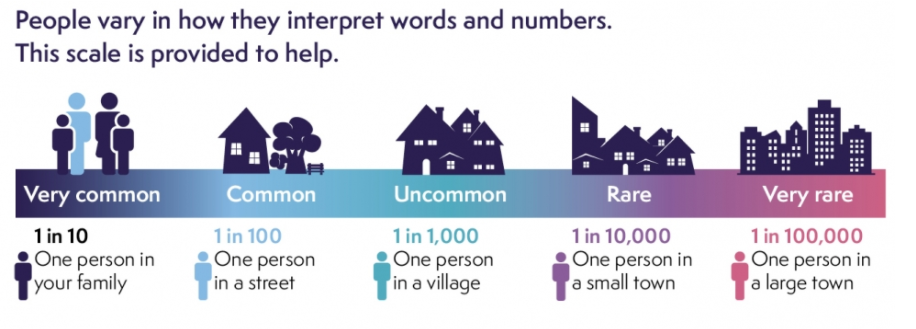

Supplement: Multimedia Appendix 1 [file mededu-v11-e70766-s001.docx]
