# Supplementary material for: Application of AI Communication Training Tools in Medical Undergraduate Education: Mixed Methods Feasibility Study Within a Primary Care Context
Source: JMIR Med Educ. 2025 Oct 24;11:e70766. doi: 10.2196/70766 (PMC12551969; doi:10.2196/70766)
Supplement: Multimedia Appendix 3 [file mededu-v11-e70766-s003.pdf]

AI Virtual Patient Focus Group Discussion Guide/Prompts for facilitator

Introduction (5 minutes)

Welcome and purpose explanation. Establish ground rules for open discussion. Promote student involvement with GP tutors present.

Educational Value and Learning Experience (10 minutes)

How would you describe your overall experience with the AI patient consultation today? What aspects were most helpful for your learning and how did it compare to practicing with real patients or student role-play? Did the AI patient prompt you to consider things you might not have thought of otherwise? How did the learning environment feel in terms of safety?

Realism and Authenticity (10 minutes)

To what extent did the AI patient feel like a real consultation? What made it feel realistic or unrealistic? How was the patient's voice, tone, and overall communication style? Did the patient respond appropriately to your questions and explanations? How well did the patient handle complex information you provided and did the patient's agenda feel authentic?

Technical Aspects and Usability (8 minutes)

How easy was the technology to access and use? What technical issues did you encounter and how did response delays affect the consultation flow? Would you feel confident using this technology independently for practice?

Areas for Improvement (10 minutes)

What would make this experience more realistic or educational? What additional patient characteristics or complexity would be helpful? How could the scenarios be enhanced and what other clinical situations would benefit from this technology? Consider patient emotions, multiple agendas, challenging behaviors, and scenario variety.

Future Applications (8 minutes)

How do you see this fitting into your medical education going forward? What would make you want to use this for independent practice? How could this support OSCE preparation or assessment and where else in the curriculum might this be valuable?

Closing (5 minutes)

Any final comments or suggestions for improvement?
